# Intraductal Papillary Mucinous Neoplasm of the Pancreas: An Update

**DOI:** 10.6064/2012/893632

**Published:** 2012-11-28

**Authors:** Shu-Yuan Xiao

**Affiliations:** Department of Pathology, University of Chicago Medical Center, 5841 South Maryland Avenue, MC6101, Chicago, IL 60637, USA

## Abstract

Intraductal papillary mucinous neoplasm (IPMN) is a cystic tumor of the pancreas. The etiology is unknown, but increasing evidence suggests the involvement of several tumorigenesis pathways, including an association with hereditary syndromes. IPMN occurs more commonly in men, with the mean age at diagnosis between 64 and 67 years old. At the time of diagnosis, it may be benign, with or without dysplasia, or frankly malignant with an invasive carcinoma. Tumors arising from the main pancreatic duct are termed main-duct IPMNs, those involving the branch ducts, branch-duct IPMNs. In general, small branch-duct IPMNs are benign, particularly in asymptomatic patients, and can be safely followed. In contrast, main-duct tumors should be surgically resected and examined carefully for an invasive component. In the absence of invasion, patient's survival is excellent, from 94 to 100%. For patients with an IPMN-associated invasive carcinoma, the prognosis overall is better than those with a *de novo* pancreatic ductal adenocarcinoma, with a 5-year survival of 40% to 60% in some series. However, no survival advantage can be demonstrated if the invasive component in an IPMN patient is that of the conventional tubular type (versus mucinous carcinoma). Several histomorphologic variants are recognized, although the clinical significance of this “subtyping” is not well defined.

## 1. Introduction

In the early 1980s, it became clear that while some mucinous cystic tumors of the pancreas do not communicate with the pancreatic duct system, many others arise within the duct. The latter are unique biologically, pathologically, and clinically, although they were traditionally “lumped” into the category of mucinous cystadenomas (or cystadenocarcinomas if malignant features were identified). The term “intraductal papillary mucinous tumor (IPMT)” and several other terms (including adenoma and carcinoma) were proposed to describe these intraductal lesions. These tumors were “officially” separated from mucinous cystadenoma in the 1996 World Health Organization (WHO) Classification. Currently, the preferred name is *intraductal papillary mucinous neoplasm* (IPMN). 

When invasive carcinomas arise from IPMN, overall they are associated with better prognosis and patient survival as compared to carcinomas not associated with IPMN. In this paper, clinical and pathology characteristics, with emphasis on update in classification and risk assessment, are discussed, based on extensive literature review and the author's experience. Attempt is also made to address several questions regarding the biologic behavior of these tumors and invasive carcinomas arising from them. 

## 2. Definition

IPMN is a grossly and radiographically discernible cystic tumor, involving the pancreatic duct system. Most tumors have a papillary intraluminal growth, causing dilatation of the involved duct and its proximal segment. The criteria for judging ductal dilation are diameters of more than 5 mm and 3 mm for the main and branch ducts, respectively. The typical tumor has a papillary intraluminal growth. With extreme dilation, the papillary nodule may become inconspicuous in some cases. There is a clear tendency for these lesions to become invasive carcinoma, in the form of either a colloid carcinoma or conventional tubular-type adenocarcinoma. 

## 3. General Features

Since its first description in the early 1980s, more cases of IPMN have been diagnosed yearly, although this tumor had certainly been encountered before 1980 [1]. The true incidence is unknown. A prevalence study of the Olmsted County, Minnesota population showed an incidence of 2.04 cases per 100,000 [2]. The increase in number of cases over the years is likely due to increased awareness of this entity by clinicians and pathologists, and better diagnostic and imaging techniques, since many cases are now diagnosed asymptomatically. Furthermore, while there is a 14-fold increase in incidence of IPMN as demonstrated in a recent study, IPMN-related or overall pancreatic cancer mortality remained steady [3]. Intuitively, as one of the premalignant lesions to invasive adenocarcinoma, early and increased diagnosis should have led to the decreased mortality to pancreatic cancer. Lack of such reduction in tumor-related death [3] is thus puzzling. 

IPMN seems to occur more frequently in men [4–6]. The mean age at diagnosis is reported to be between 64 to 67 years [1, 6–8]. When symptoms are present, they may include episodic pancreatitis-like symptoms, abdominal pain, jaundice, or weight loss [1, 7–9]. The symptoms are likely due to partial or complete ductal obstruction complicated by obstructive pancreatitis. Most tumors are located in the head of pancreas [7], although body and tail involvements are not uncommon. 

Microscopically, IPMNs exhibit various degrees of dysplasia in tumor epithelial cells, as will be discussed in more details below. It must be pointed out that many published studies on biological behavior and clinical outcome of IPMNs, particularly the earlier ones, included a spectrum of lesions ranging from minimal dysplasia, severe dysplasia but noninvasive, to frankly invasive carcinomas. Evidently this approach led to contradictory findings. More meaningful conclusions should be drawn on noninvasive and invasive tumor separately. 

## 4. Etiology, Pathogenesis, and Molecular Abnormalities

The etiology of IPMN is unknown. Currently, there is insufficient data to propose a putative pathogenesis for these tumors. Summarized in the following paragraphs are rather “fragmented” findings from studies of different molecular targets. 

 Similar to other tumors of the gastrointestinal tract, but less frequently, IPMNs occur in settings of a genetic cancer syndrome, including familial adenomatous polyposis (FAP) or attenuated FAP [10, 11], familial pancreatic carcinomas [12–14], and Lynch syndrome [15]. In a report of a 48-year-old man with a history of FAP and an IPMN, genetic analysis revealed loss of the wild allele of the APC gene in the tumor, leading to inactivation of both alleles [10]. This provided direct evidence of association between IPMN and FAP. Furthermore, involvement of the Wnt-signaling pathway had been demonstrated in some cases of IPMN with high grade dysplasia or invasive carcinoma [16]. Abnormal beta-catenin nuclear staining was reported in 40% of cases, half of which were accompanied by loss of APC protein [16]. Rarely IPMNs occur in patients with Lynch syndrome, accompanied by lack of MSH2 and MSH6 expression, and microsatellite instability (MSI) phenotype in the tumors [14, 15]. IPMN had also been described in association with Peutz-Jeghers syndrome (PJS) [17]. Rarely IPMN may occur in first-degree relatives without other familial syndromes [18].

On another hand, concurrent or metachronous extrapancreatic neoplasms are not uncommon in patients with IPMN [14, 19–22]. The reported rates range from 10% [20] to 32% [23–25] in different studies. More interestingly, the rate of extrapancreatic malignant tumors is reported to be higher in patients with IPMN than those with PDAC [14]. The observed extrapancreatic neoplasms include colonic adenoma or carcinoma [14, 19], gastric [19], bile duct [23], breast and prostate carcinomas [20, 21].

Occurrence of IPMNs in patients with a familial cancer syndrome and their association with extrapancreatic neoplasms suggest that tumorigenesis of IPMN share molecular pathway abnormalities seen in these other tumors. Pathogenetically, many risk factors identified for IPMN seem to overlap with that of pancreatic ductal adenocarcinoma (PDAC) [26]. 

### 4.1. Copy Number Alteration

In a study of 20 IPMNs using array comparative genomic hybridization (aCGH), cytogenetic alterations were identified in high grade dysplasia or invasive lesions [27]. These include regional loss in chromosome 5q, 6q, and 11q, which are different from those identified in PDAC. Loss of chromosome 6q represents the most frequently observed large-scale chromosomal aberration in IPMN. Notably, no tumor suppressor gene had been identified in this region [27]. Since in most familiar cancer syndromes a tumor suppressor gene inactivation is involved, findings like this are rather unusual. In another study of genome-wide allelotypes of familial pancreatic adenocarcinomas and IPMN, it was found that in contrast to the high frequency of LOH (average fractional allelic loss (FAL) of 50%), IPMN exhibits a much lower level of FAL (10%), and the most common locus of LOH in IPMN is 19p [12]. By interface cytogenetic analysis, a progressive genomic alteration was detected from mucinous hyperplasia, IPMN, to invasive carcinoma, involving losses of chromosome 6, 17, or 18 [28]. A more recent study using SNP array also revealed the low incidence of somatic copy number changes in IPMN from individuals with familial history of pancreatic cancer [29]. Of 8 microdissected IPMN lesions, only one showed identifiable copy number changes (loss of 9p), supporting the concept that there is no one tumor suppressor gene responsible for development of IPMN.

### 4.2. Gene Mutation

#### 4.2.1. p53

 As seen in other types of neoplasms, p53 mutation is one of the most common changes observed in IPMN [30], which is often identified as abnormal immunostain [31, 32] and/or by mutational analysis. In well-controlled studies, abnormality of p53 status is only seen in high grade dysplasia and invasive carcinoma derived from IPMN [31–34]. There is no correlation between abnormal p53 status and *k-ras* mutation in these tumors [33]. 

#### 4.2.2. DPC4/SMAD4

In IPMN without invasive carcinoma, LOH at the SMAD4 locus is frequent. However, inactivation mutation of this gene has not been observed [8, 35]. In contrast, loss of DPC4 expression can be identified in 16% of invasive carcinomas arising from IPMN, suggesting that SMAD4 mutation is rather a late genetic change in pancreatic carcinogenesis in setting of IPMN. Biankin et al. had shown loss of DPC4/SMAD4 in 3/8 intraductal papillary mucinous carcinomas (4 *in situ* and 4 invasive carcinoma cases) [36]. These authors later reported in another study the same changes in PanIN lesions and stated that these represented changes typical of conventional PDAC, and differed from IPMN in the same specimens [37]. These seemly conflicting data illustrates the need for clear pathologic description and specific lesions used in studies, before molecular studies can be carried out and data analyzed.

#### 4.2.3. *k-ras*

This is a commonly observed change, seen in 30 to 82% of IPMN [32, 38–41]. The mutations are limited to codon 12 of exon 1 [34, 38, 42–45]. The large range of reported *k-ras* mutation frequency in IPMN is likely due to the fact that studies included different proportion of cases with an invasive component. As well known, the latter exhibit higher frequency of *k-ras* mutation than those without invasion [32, 42]. In fact, in conventional invasive pancreatic ductal adenocarcinomas (PDAC), *k-ras* mutation rate is nearly 100%. Furthermore, the frequency of mutation also differs among histologic subtypes [45]. Analytical specificity also accounts for the different frequencies. For example, a study reporting a 71% *k-ras* mutational rate in IPMN also found the same mutations in 42% of chronic pancreatitis lesions [39]. 

Some investigators interpret the difference in overall *k-ras* mutation frequency (lower for IPMN as compared to that of PDAC) as to suggest a fundamental difference in tumorigenesis between these two tumors. This comparison may not be entirely relevant, since the former encompasses a spectrum from benign to invasive cases and the latter are all invasive carcinomas. As well known, *k-ras* mutation occurs in different frequency between PDAC and pancreatic intraepithelial neoplasms (PanIN), another premalignant lesion. By analogy, the difference between IPMN and PDAC in terms of *k-ras* mutation rate simply reflects the fact that most cases of IPMN examined in these studies are premalignant lesions and may not reflect fundamentally different molecular pathways in most cases. A more meaningful and proper comparison should be done between invasive carcinomas arising from IPMN, and those not. Further supporting this notion, it had been shown that frequency of *k-ras* mutation increases with grade of dysplasia in IPMN lesions [38]. In a hamster model of chemically induced pancreatic adenocarcinomas and IPMN, *k-ras* mutation had been identified in similar frequency in both [46].

A field cancerization effect (cancer “field effect”) seems to play a role in at least some cases of IPMN [47]. Studies examining separate lesions of IPMN and hyperplasia in the same pancreas had shown distinct *k-ras* mutations, suggesting multifocality of the tumor may originate from multiple precursor lesions [47]. 

#### 4.2.4. BRAF

 BRAF mutations have been noted in cases of IPMN as well [43, 44, 48]. However, this seems to be rare and not a main genetic event involved in IPMN tumorigenesis [45]. 

#### 4.2.5. Phosphoinositide-3-Kinase Catalytic-Alpha (PIK3CA) and Akt

 Phosphoinositide-3-kinase catalytic-alpha (PIK3CA) mutations are involved in several tumors. Some of these mutations activate the *Akt* signaling pathway. One of its component, Akt/PKB, is believed to promote cellular proliferation and inhibit apoptosis. PIK3CA mutations had been identified in some cases of IPMN as well [32, 44, 45]. The frequency of these mutations appears to be greater in the tubulopapillary subtype [45], which is related to increased phosphorylated *Akt*. Another immunohistochemical study showed overexpression of phosphorylated *Akt* (activation) in 63% (10/16) of IPMNs, similar to that of PDAC (70%) [49]. 

#### 4.2.6. CDKN2A-p16

Mutation of this tumor suppressor gene is involved in tumorigenesis of pancreatic adenocarcinomas, leading to loss of p16(INK4A). The latter had been shown immunohistochemically in IPMN with high grade dysplasia or invasion as well [36]. The functioning isoforms of this gene interact with MDM2 in stabilizing p53, and to inhibit CDK4.

#### 4.2.7. GNAS

More recently, by analyzing cystic fluid DNA, Wu et al. had identified mutation of GNAS in up to 66% of IPMNs, which are lacking in other cystic lesions [41]. Many of these cases also show *k-ras* mutations. This finding may have potential in preoperative differential diagnosis between a nonneoplastic cyst and IPMN. However, there seems to be no difference in frequency of GNAS mutation between main duct and branch duct IPMNs, or between benign and invasive lesions (Ellsworth E. M. et al. “*The role of GNAS mutation in diagnosis of mucinous pancreatic cysts.*” Abstract presented at the 2012 ASCO Annual Meeting). 

#### 4.2.8. Other Mutational Abnormalities

 BRG1 is a component of chromatin remodeling complex SW1/SNF regulating transcription (subunit BRG1/SMARCA4) and is inactivated in several other malignancies. Loss of BRG1 expression had been demonstrated in up to 50% of noninvasive IPMNs as well [50], and the status of BRG1 loss did not correlate with histologic types. 

Using an antibody for Thr (68)-phosphorylated chk2, decreasing expression toward higher atypia had been shown in IPMNs [31], suggesting the involvement by DNA damage checkpoint pathway, along with increasing p53 accumulation. In addition, some studies had suggested roles played by sonic hedgehog signaling pathway in IPMN tumorigenesis [51, 52]. 

Other molecular abnormalities had been observed in IPMN and may have potential to be used as biomarkers to help identifying precursor lesions. These include deleted in malignant brain tumor 1 (DMBT1) and tissue transglutaminase 2 (TGM2), which are overexpressed in IPMNs [53]. C-erbB-2 overexpression is identified in 65% of IPMN associated with dysplasia [33]. Whole-exome sequencing also uncovered somatic mutations in KCNF1, DYNC1H1, PGCP, and several other genes [54]. 

#### 4.2.9. STK11/LKB1 Peutz-Jeghers Gene Inactivation

As a tumor suppressor gene, STK11/LKB1 abnormality is the underlying mechanism for Peutz-Jeghers syndrome (PJS). Using PCR to amplify 5 microsatellite markers from the 19p13.3 region (harboring the STK11/LKB1 gene), LOH of this region was identified in 5 of 20 (25%) IPMNs arising in patients with PJS [17]. However, it is not clear if mutation of this gene is directly responsible for IPMN. 

### 4.3. Epigenetic Alterations

By using global expression profiling and RT-PCR, Sato et al. identified epigenetic downregulation of the cyclin-dependant kinase inhibitor, CDKN1C/p57KIP2, in IPMN, which is also present in many pancreatic cancer cell lines [55]. Further studies reveal that CDKN1C is commonly downregulated in pancreatic ductal neoplasms through promoter hypermethylation and histone deacetylation [55]. Promoter methylation of at least one tumor suppressor gene can be demonstrated in IPMN [56], particularly p16 and p73; and IPMN with invasive carcinoma showed a higher rate of aberrant tumor suppressor gene methylation. CpG island hypermethylation of selected genes had been observed in some IPMN lesions, some related with high grade dysplasia such as BNIP3 [57]. 

### 4.4. MicroRNA

Significant fold-increase of miR-155 and miR-21 had been demonstrated in IPMN, with similar increase in pancreatic juice samples of miR-155 by quantitative RT-PCR [58]. 

In addition to direct observations in patient tumors described above, experimental evidence of involvement by some of the above molecular abnormalities also starts to accumulate. Siveke et al. established a mouse model by crossing Elastase-Rgfa mice with p48 (+/Cre); Kras (+/LSL-G12D) mice, in which concomitant expression of TGF-alpha and *k-ras* (G12D) led to development of cystic lesions resembling IPMN [59]. In another mouse model, combination of *K-ras* (G12D) and SMAD4 deficiency was found to lead to the development of IPMN [60]. SMAD4 seems to function in blocking progressing KRAS activation initiated tumorigenesis, either in PDAC or IPMN. 

## 5. Pathology of IPMN

IPMN mostly occurs from the Wirsung's duct (WD) but may also arise in Santorini's duct (SD) [61, 62]. In most patients, the lesion is located in the head of pancreas, but tumors involving the body or tail are not uncommon [8]. Involvement of the entire pancreas by tumor had been reported to occur in up to 20% of cases [46].

**Figure 1:**
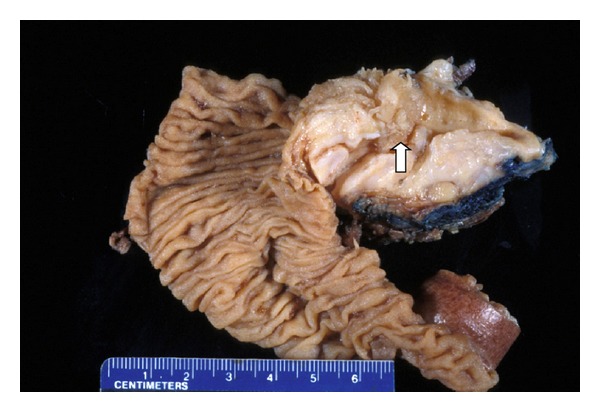
Intraductal papillary mucinous neoplasm, main duct type (arrow). Note the cystically dilated segment.

**Figure 2:**
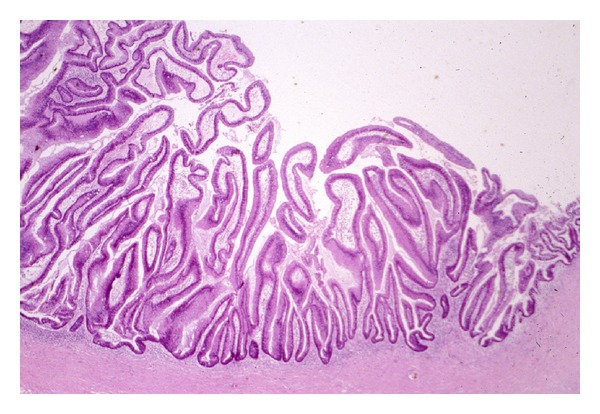
An IPMN with mostly villous structures protruding into the cyst lumen.

**Figure 3:**
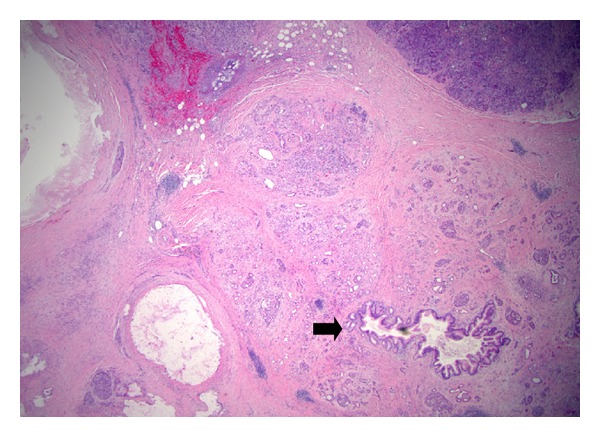
Atrophy of parenchyma associated with IPMN. The acinar parenchyma in the vicinity of an IPMN (not shown) exhibits loss of lobular acini, with a central duct and islets of Langerhans remained. Note the small duct (arrow) is secondarily involved by changes of IPMN, with microscopic papillary overgrowth of the epithelium.

**Figure 4:**
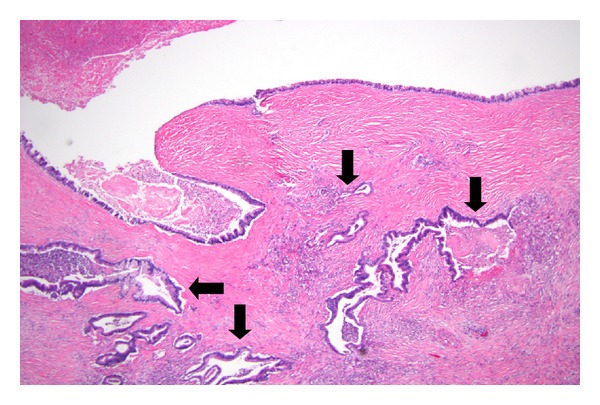
Invasion arising from an IPMN. The cystic IPMN in this field exhibits nearly flat mucosa, but invasive carcinoma as characterized by angulated abortive ducts is evident in the surrounding densely fibrotic stroma (arrows).

Typically, the pathology is that of an ectatic or cystically dilated segment of pancreatic duct, with papillary growth [30] (Figure 1). To a large extent, the tumor pathologically resembles a colonic villous adenoma, as a neoplastic epithelial growth protruding into the lumen of the duct (Figure 2). There is often progressing cytologic atypia in the lining epithelium. Some studies observed a papillary growth (intraductal adenoma) in a portion cases (4 of 14 cases examined in one study [5]). Due to the intrinsic nature of small caliber of the pancreatic ducts, obstruction and dilatation are inevitable in most cases. In a subpopulation of them, with prominent cystic dilatation, a luminal papillary lesion may become inconspicuous due to exposure to the increased intraluminal pressure. In addition, obstruction of the duct also causes fibrotic atrophy of the surrounding nontumor parenchyma [4]. Thus, microscopically, pancreatic parenchyma close to the dilated ducts usually show mild to moderate fibrosis and acinar atrophy typical of obstructive chronic pancreatitis (Figure 3). Some tumors eventually progress to invasion into the subepithelial stroma, to form an invasive carcinoma (Figure 4). Occasionally, focal to extensive calcification may lead to changes of the so-called calcifying obstructive pancreatitis [63], but calcification usually does not involve the tumor itself.

IPMN may be multifocal, either synchronous or metachronous [64]. The majority of these cases are of branch duct type (see below) and exhibit gastric-foveolar epithelium. They often show independent genetic alterations among multiple lesions in the same pancreas, including *k-ras* mutation, and LOH profiles [64].

### 5.1. Main Duct and BD-IPMN (or Side-Branch (SB) IPMN)

**Figure 5:**
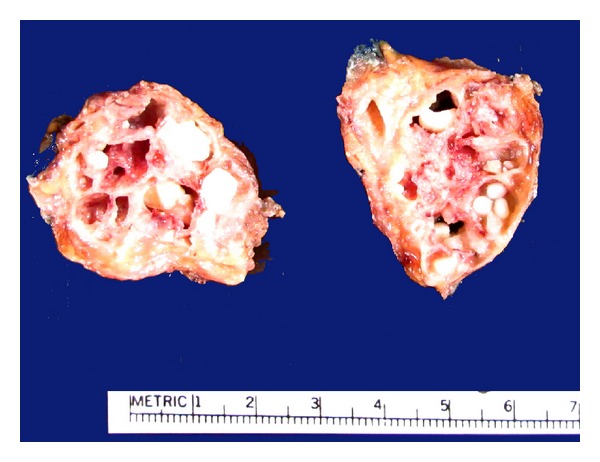
Branch duct IPMN. The normal pancreatic parenchyma in this location is replaced by the multicystic tumor. Many of the cysts contain inspissated mucous, with calcification.

**Figure 6:**
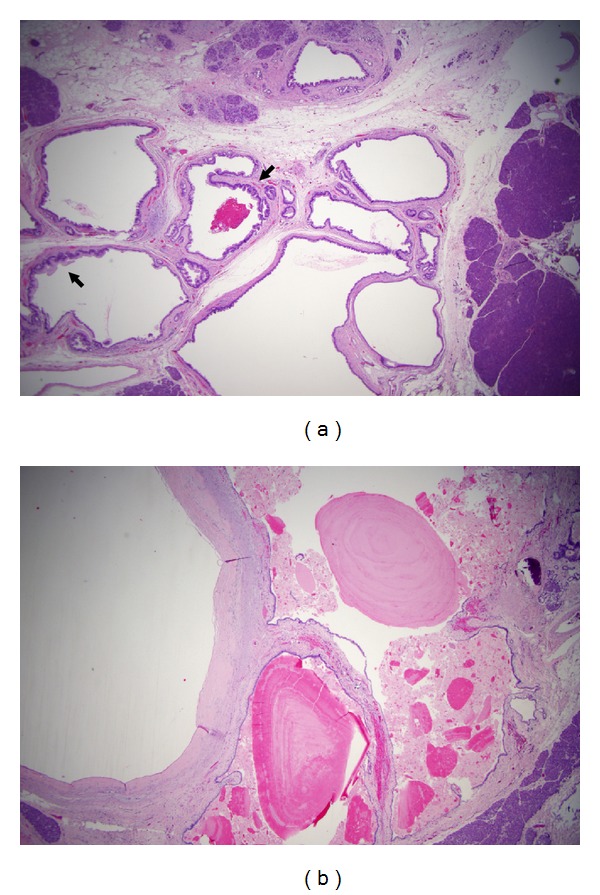
Branch duct IPMN. (a) The tumor is characterized by clusters of micro- and macrocysts, with lining epithelia focally showing proliferative growth (micropapilli) (arrows). (b) Other cysts show near-total flattening of lining epithelium, or loss of epithelium, with eosinophilic inspissated mucin.

IPMNs can be placed into one of two main types based on the segment of ductal system they arise from: main duct (MD)-IPMN and branch ducts (BD)-IPMN [65] (Figure 5). The latter is also designated side branch (SB)-IPMN by some investigators. Some tumors can involve both and thus are designated mixed type. Overall, BD-IPMNs constitute about 30 to 39% of all IPMNs [65, 66]. In a study of 44 cases, nearly all BD-IPMNs were located in the head of pancreas, whereas 33% of main duct cases involved the tail or body [65]. In addition to the distinct anatomic locations in the pancreas, these two types of tumor also differ from each other in risk of disease progression, and pathology. Typical MD-IPMN presents as a nodule/mass lesion in a dilated duct or cyst (Figure 1). In contrast, many BD-IPMNs are multicystic, lack nodular formation, and commonly contain inspissated mucin material (Figures 5 and 6). In contrary to what the name suggests, a papillary component is usually not a part of the lesion in BD-IPMN (Figure 6). Most branch duct tumors show no or low-grade dysplasia, and rarely with focal high-grade dysplasia (CIS). One study showed 15% rate of carcinoma* in situ* in BD-IPMNs [65]. For main duct IPMN, 37% contained an invasive carcinoma [65].

### 5.2. Histologic Subtypes and Grade of Dysplasia

**Figure 7:**
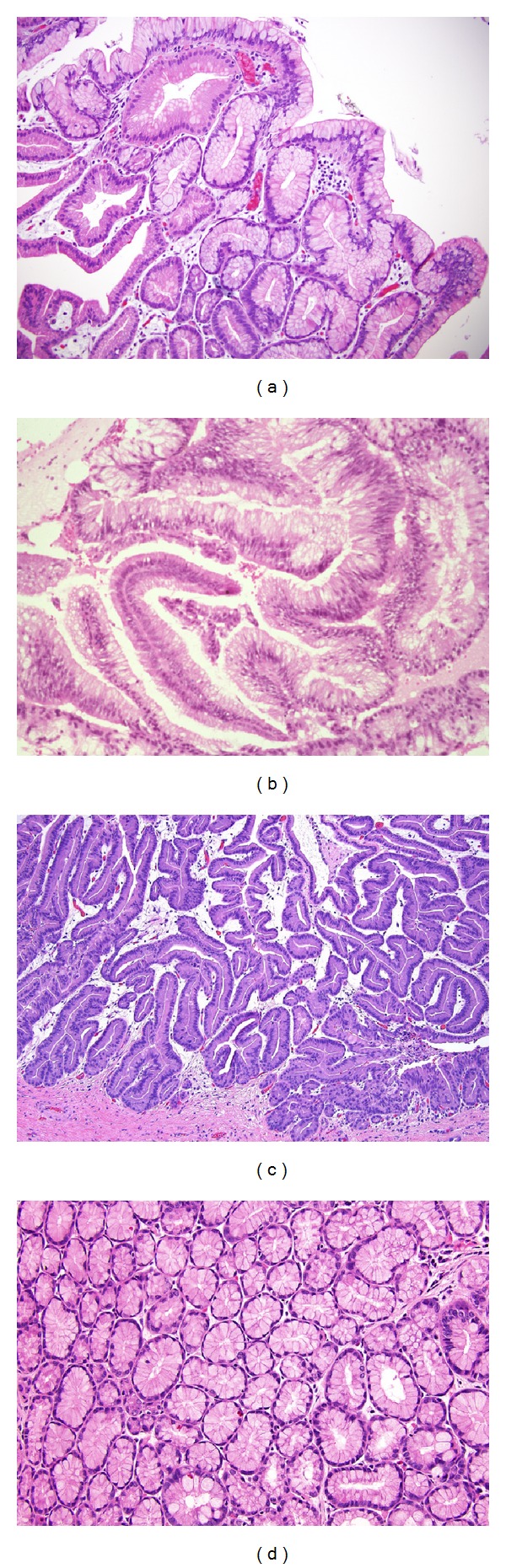
Histologic subtypes of IPMN based on histomorphology of the epithelia. (a) Foveolar subtype, mimicking gastric foveolar epithelium, with no or minimal dysplasia. (b) Intestinal subtype, with low grade dysplasia. (c) Pancreatobiliary subtype, with moderate dysplasia. (d) Pyloric gland adenoma subtype. No dysplasia.

Histologically, IPMN exhibits one of several types of epithelium, namely, gastric foveolar, intestinal, pancreatobiliary, oncocytic [67, 68], and tubulopapillary [69] (Figure 7). The prevailing component of epithelium is used to assign a tumor to a corresponding histomorphologic subtype. Therefore, it is common to see an intestinal or pancreatobiliary subtype mixed with focal foveolar epithelium. Resembling colonic tubulovillous adenomas, the intestinal type are characterized by tall columnar epithelia with elongated nuclei, with goblet cells (Figure 7(b)). The pancreatobiliary type is characterized by arborizing papillae lined by cuboidal cells resembling papillary neoplasm of the biliary tract (Figure 7(c)). Focal cribriform changes may be prominent. Coincidently, these cytologic features are also related to morphology used for grading dysplasia. Therefore, foveolar subtype lesions usually are of no or minimal dysplasia, as the epithelial cells exhibit abundant cytoplasm and small basally arranged nuclei (Figure 7(a)). Intestinal subtype lesions often exhibit mild dysplasia (Figure 7(b)), and pancreatobiliary subtype, moderate to severe dysplasia (Figure 7(c)). In intraductal oncocytic papillary neoplasm, the tumor cells are characterized by plump abundant eosinophilic cytoplasm, which immunohistochemically stain with antibody for mitochondria [70]. 

Therefore, classifying tumors based on epithelial morphology may to certain extent have clinical significance. Some authors claim that histologic subtype is the second most significant predictor of survival, after staging [68]. In this multicenter analysis of 283 surgically resected IPMNs, it was found that patient survival (Kaplan-Meier curve) at 5 and 10 years were both .937 for gastric-type; 0.888 and 0.685 for intestinal type; 0.839 and 0.734 for oncocytic-type; 0.52 and undetermined for pancreatobiliary type (insufficient number of cases). 

**Figure 8:**
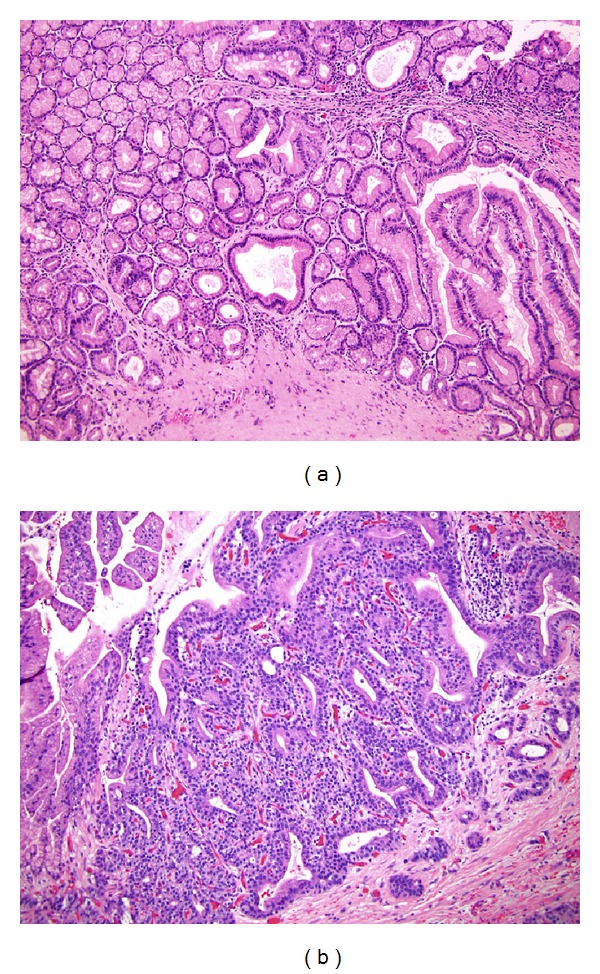
Mixed components in IPMN. Both of these lesions show features of the so-called tubulopapillary subtype. (a) The left half to the pictured field contain pyloric gland adenomatous component, while the right side show foveolar configuration. Also note the scattered goblet cells mostly in the foveolar component. (b) The tubular component exhibits high grade dysplasia. An invasive ductal adenocarcinoma arose from this lesion (not shown).

Occasionally, either independently or in association with an otherwise typical gastric type IPMN, a pyloric gland-type lesion occurs, which is designated intraductal tubular adenoma by some [71–73] (Figure 7(d)). Histologically and immunohistochemically, this tumor resemble the pyloric adenoma of the gallbladder. But they otherwise show similar features of IPMN, except for the lack of prominent papillary structures. However, as described above, losing papillae is not uncommon in IPMN and should not be ground for considering these lesions as a separate entity. Alternatively, this type of tumor may be considered a variant of the foveolar type IPMN. In the author's experience, focal pyloric gland component is not uncommonly seen in an otherwise typical gastric foveolar subtype of IPMN (Figure 8). In addition, in concordance with their nondysplastic property, the cells do not show nuclear immunostaining for p53, or loss of DPC4 [72]. Others have used the term “intraductal tubulopapillary neoplasm” to describe cases in which the intraductal tumor is characterized by solid mass without evident mucin [69]. However, all cases exhibited positive MUC1 immunostain, supporting the mucinous nature of this lesion. It may be reasonable to consider this yet another morphologic variant of IPMN, rather than a separate disease, although most lesions are characterized by high grade dysplasia.

Of interest is that gastric foveolar-type IPMNs often are found in BD-IPMN (98%), whereas intestinal type usually in main duct IPMN (73%) as shown in one study [74]. Furthermore, the intestinal type is also more frequently associated with severe atrophy and fibrosis in the surrounding parenchyma with mucus lake formation. In this same study, 23% of the intestinal type lesions are associated with an invasive carcinoma and only 2% of gastric type had invasive ductal carcinoma [74].

Pathogenesis leading to different histologic subtypes is unknown. Some speculate that these subtypes represent unique pathways of tumorigenesis [45, 75]. However, it is also possible that they simply represent a dynamic metaplastic process after initiation of IPMN tumorigenesis, due to the unique location and spatial confinement of these tumors. As mentioned previously, it is not uncommon to find mixed histologic components in the same IPMN, representing diverging differentiation (Figure 8). But in general, there is not sufficient data to support either one of these speculations. As one of the most well-known pathologic phenomena, intestinal metaplasia is commonly associated with chronic mucosal inflammatory injury of the gastrointestinal tract, be it in the distal esophagus (Barrett's esophagus), stomach (atrophic gastritis, *H. pylori* gastritis, or severe atrophy due to chronic graft versus host disease). Similarly, peptic duodenal injury is often accompanied by gastric foveolar metaplasia. Stricture, chronic ulceration of small intestine is known to cause gastric pyloric gland metaplasia, such as in Crohn's enteritis. It may be that as some IPMNs reach certain size and causes chronic obstruction, increased intraluminal pressure and epithelial injury initiate the metaplastic process, either intestinal or gastric foveolar type. In contrast, colonic adenomas, being small tubular or large villous adenoma, rarely undergo metaplastic changes, perhaps due to the much larger luminal space they enjoy. Hypothesis aside, current observation does point to a loose association between histologic subtype and degree of dysplasia, as previously mentioned.

Interestingly, recent studies have started to show that the histologic subtypes of IPMN exhibit variation in molecular pathway involved. For example, gastric subtype has a higher frequency of KRAS mutation as compared to the intestinal subtype (82% versus 27%), and lower incidence of SMAD1/5/8 phosphorylation (27% versus 82%) [75]. The tubulopapillary type exhibits a higher frequency of PIK3CA mutation and AKT phosphorylation [45], as compared to other subtypes. Nevertheless, additional studies that are more inclusive in number and type of cases, and more systematic, are needed before conclusions can be drawn. 

### 5.3. Invasive Carcinoma Arising from IPMN

Some investigators estimate that 15 to 40% of cases of IPMN are associated with invasion at presentation [4, 8, 33]. Among resected specimens performed for a preoperative diagnosis of IPMN, 19 to 24% contain an invasive component [33, 76, 77]. Another study involving 136 pancreatic resections for IPMN showed that 38% cases had an invasive component [78]. Lymph node metastasis had been identified in 20% [33] to 54% [78] of cases with an invasive component. Some of the factors associated with an invasive component in resected IPMNs include larger tumor size and main-duct involvement [6].

**Figure 9:**
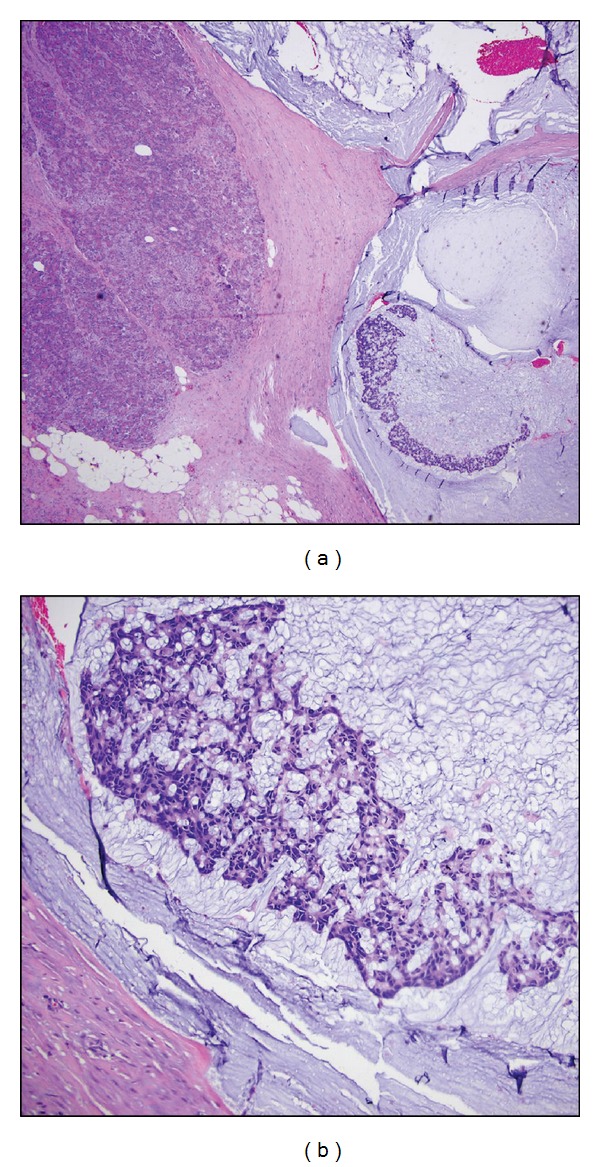
Colloid carcinoma or mucinous noncystic carcinoma arising from an IPMN. (a) Large mucin “lakes” representing invasive adenocarcinoma replacing normal pancreatic parenchyma (residual parenchyma in the left edge of the picture). Clusters of tumor epithelial cells are seen “floated” in the mucin, as seen in higher magnification in panel (b).

When invasive carcinomas develop in IPMN, one of two main histologic types can be encountered, namely, colloid carcinoma (Figure 9), or the tubular adenocarcinoma (Figure 4), the latter is indistinguishable from the usual ductal adenocarcinoma arising in patients without IPMN. Colloid carcinoma is also designated mucinous noncystic carcinoma and had been suggested by some authors to be the more prevalent type associated with IPMN [5]. However, other systemic studies had suggested a higher frequency of tubular carcinoma than colloid carcinoma [79]. Other types, such as an oncocytic type, had also been seen [80]. This later study comprising 61 IPMN-associated invasive carcinoma identified the usual tubular type in 62%, colloid in 26%, and oncocytic in 12% of cases [80], making the tubular type the most frequent type. 

A category of minimally invasive IPMN had been described in a Japanese classification but not in the WHO classification. A recent study attempting to better define the diagnostic criteria showed that cases classified as minimally invasive had identical outcome as those of noninvasive cases [81], raising the question if these represented true invasion from a biological point of view. The diagnostic criteria listed were rather cumbersome and poorly defined, making reproducibility challenging. For example, mucus rapture and duct expansion were treated as minimally invasive. But these are more likely results from effect of high intraluminal pressure caused by obstruction, similar to that seen in appendiceal mucinous cystadenoma [82]. One of the “objective” criteria is invasion less than 5 mm [81], but without an explicit morphologic definition as depicted in the figures in the paper, and some of the lesions described as invasive appear to be direct involvement of the tributary ductules by IPMN. Therefore, it seems that introducing this category into our diagnostic practice currently lacks sufficient scientific support and will likely lead to more unnecessary complexity in clinical management. Therefore, use of this terminology should be discouraged until more evidence become available to support its significance and objective identification. 

### 5.4. Concurrent Pancreatic Endocrine Tumors

Occasionally both a pancreatic endocrine tumor (PET) and IPMN may be identified, from pancreas resected either for PET or IPMN. In one report, 6 PETs were identified in 103 cases of IPMN [83], giving rise to a frequency of 5%. The significance of this phenomenon is currently undetermined, before additional data become available. 

### 5.5. Immunohistochemistry

Naturally, the epithelia of all IPMN subtypes stain positive for pan-cytokeratins immunohistochemically. However, depending on the histologic subtype, unique profile for specific cytokeratins and mucin-protein markers is observed in individual tumors. Overall, most mucinous metaplastic lesions express MUC1 [84], including pancreatic intraepithelial neoplasms (PanIN) and IPMN [85]. Except for the intestinal type, IPMNs in general are negative for CDX2, CD10, and CK20 [86]. Gastric-type IPMNs exhibit expression of MUC5AC, but expression of MUC6 is variable [87]. The pancreatobiliary type lesions are positive for MUC1, weak but diffuse positive for MUC6. The intestinal type lesions are uniformly positive for CDX2, MUC2, but some also express MUC5AC focally. The oncocytic lesions mostly express MUC1 and MUC6 [87]. It should be noted that scattered goblet cells can be seen in many of the nonintestinal subtypes of IPMN, therefore will render the tumor focally positive for MUC2, CDX2, and CK20. The vast majority of invasive intraductal papillary mucinous carcinomas (IPMC) and ductal adenocarcinoma express MUC1 as well, in 86% and 100% cases, respectively, supporting the notion that pancreatobiliary type lesions carry a much greater risk for malignant transformation. Colloid carcinoma expresses the intestinal-type markers (MUC2). Gastric type markers are seen in noninvasive tumors more frequently [33, 88], corresponding to lower rate of dysplasia associated with the latter. 

For intestinal type, expression of another marker for intestine-specific marker, liver-intestine cadherin (LI cadherin) is increased both immunohistochemically, and at the mRNA level. The level of expression seemed to correlate with CDX2 and exhibit gradual increase with degree if dysplasia [89]. In foci of severe dysplasia or invasive carcinoma, abnormal nuclear staining of p53 is common [30, 90], as described above. Similarly, increased proliferative activity can be demonstrated by immunostaining for PCNA and Ki-67 at these foci [30, 90, 91]. There seems to be a gradual increase in Ki-67 index according to the grade of dysplasia in IPMN as well [90]. 

By analyzing microdissected tumor cells using quantitative RT-PCR, increased expression of S100A2 is found in invasive pancreatic adenocarcinomas, in contrast to IPMN [92]. But increased expression of S100A1 and S100P had been observed in both PDAC and IPMN [93, 94]. 

Loss of expression of claudin-1 and claudin-4 had been reported in IPMN and carcinoma [95]. Also, 22% of IPMN (19 of 88 cases studied) show loss of staining for SOX17 [57]. The diagnostic value of these stains had not been examined.

Due to association with FAP, some cases of IPMN can have abnormal nuclear stain for beta-catenin, sometimes with concurrent loss of APC protein [16].

Some immunohistochemical (IHC) targets have also been examined for their value in identifying malignant lesions. For example, Plec-1 is found to be positive in 26 of 31 malignant IPMN (high grade dysplasia or invasion) but 1 in 6 benign IPMN [96].

CD133 is normally expressed in the centroacinar region and intralobular duct cells, as well as ductal adenocarcinomas, but negative for cells of IPMN or IPMN-associated carcinomas. Although S100A4 [97] and S100A6 [98] had been found to show increased expression in pancreatic adenocarcinomas, it is not the case for IPMN. Expression of MSX2 had been suggested to be an independent predictive factor for malignancy in IPMN [99]. Expression of KOC in cytology may be of value to mark PDAC, if using of 3+ staining intensity in more than 75% of cells as cutoff [100].

As evaluation of invasion can be challenging, immunostain for certain basement membrane component had been studied. Type IV collagen alpha chains may be lost in association with invasion [101]. However, due to complex combination of different alpha chains, staining for each individual type of alpha chain may be required, rendering this marker impractical. 

### 5.6. Distinction between IPMN and PanIN

**Figure 10:**
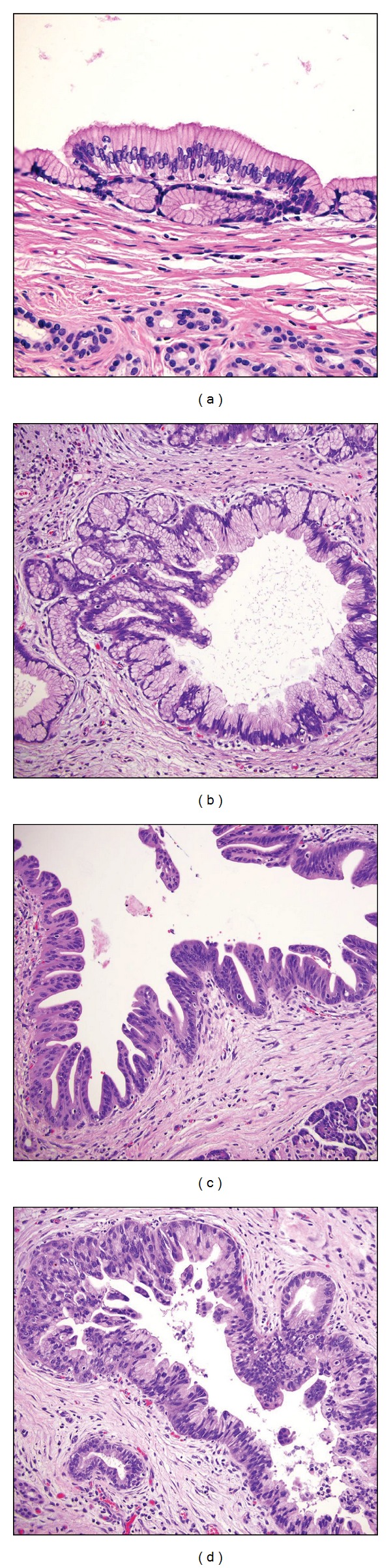
Pancreatic intraepithelial neoplasia (PanIN). (a) PanIN 1A, flat mucinous metaplasia. (b) PanIN 1B, micropapilli formation, no dysplasia. (c) PanIN 2, mild to moderate dysplasia. (d) PanIN 3, severe dysplasia.

Pancreatic intraepithelial neoplasia (PanIN) defines a *de novo* neoplastic transformation of ductal epithelium, which usually starts as focal mucinous metaplasia. The lesion is believed to undergo stepwise progression from no or minimal dysplasia (PanIN 1) to moderate dysplasia (PanIN 2) and severe dysplasia or carcinoma *in situ* (PanIN 3). Essentially, these denote lesions in small interlobular ducts that do not form macroscopically identifiable lesions (Figure 10). Whether they are biologically truly distinct from early branch duct IPMN is subject to debate. Since they are frequently associated with invasive ductal carcinoma, it is practical to consider them as a separate premalignant disease. 

For the most part, separating PanIN and IPMN seems straightforward, as by definition the former is not clinically or radiographically evident, and there is no macroscopically identifiable lesion. They are recognized as a microscopic abnormality during examination of resected specimens. However, there is significant overlap in terms of morphology and molecular abnormalities between small IPMN and PanIN. Distinction between the two can sometimes be difficult. The arbitrary nature of this distinction had been illustrated in an interobserver variation study participated by expert pancreatic pathologists from Europe, Japan, and the US, in which frequent disagreement was shown among them [102]. 

A criterion defined by a consensus agreement states that lesions less than 0.5 cm in diameter are categorized as PanIN [103]. Although its biologic basis remains to be determined, this morphometric criterion seems to improve the interobserver agreement significantly [102]. If for no other purpose, separating these two lesions in a more consistent and “objective” manner will help to make future comparative study more relevant and conclusions closer to the biological truth. Nonetheless, it should be noted that when this 0.5 cm in diameter rule is used, no adjacent IPMN lesion should be present, as the latter can involve smaller branches of the duct, mimicking a PanIN lesion. 

An argument can be made that IPMN and PanIN may be biologically related processes, but involving different regions of the duct system. It is also possible that at least some IPMNs represent PanIN involving larger ducts. Biologically, there is no reason why PanIN cannot arise anywhere in the duct system, including the main duct. Therefore, it is only logical that they may serve as precursors for IPMN, thus are morphologically indistinguishable from the latter. This probably explains the overlap in molecular pathways abnormalities found between these two lesions. Alternatively, IPMN itself may be a heterologous group of tumors, arising from different pathways (e.g., intestinal versus pancreatobiliary) as presented in some studies [74, 75, 87, 89]. This may partially explain the inconsistent findings among IPMN lesions when molecular events were examined. Therefore, future studies of IPMN pathogenesis should separate lesions of different histomorphologic types. Lumping all IPMN lesions in studies results in data that are difficult (if not impossible) to interpret. 

Except for PanIN 1 lesions, which are common manifestations in atrophic focus, including areas adjacent to an IPMN, true concurrent PanIN and IPMN are rare. Most of these represent direct extension of IPMN to smaller ductules. An arbitrary approach taken by some authors [37] to separate PanIN from extension of IPMN is the requirement of a minimum distance of 5 mm from the IPMN. PanIN lesions found in pancreas resected for IPMN show protein expressions related to tumorigenesis, in a fashion similar to that involved in conventional ductal carcinoma and may be independent from the IPMN [37]. Another study of p16INK4A and p53 immunohistochemical staining pattern showed that loss of p16INK4A expression or overexpression of p53 was more frequently seen in PanIN 3 than in IPMN with severe dysplasia [104].

### 5.7. Distinction from Mucinous Cystic Neoplasm (MCN)

**Figure 11:**
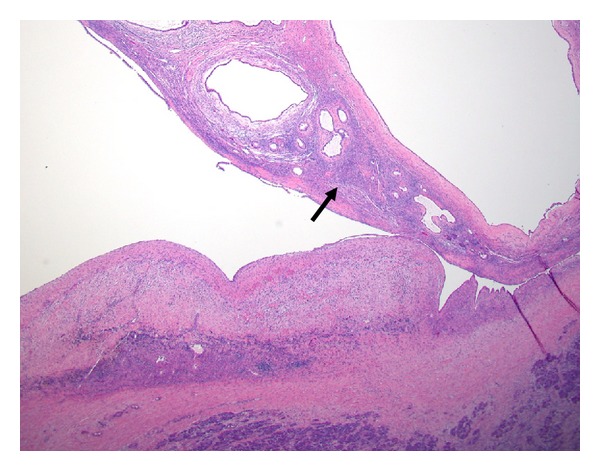
Mucinous cystic neoplasm. In this example of multicystic lesion, the lining mucinous epithelium is focally flattened. The cyst wall is characterized by the “ovarian” type mesenchymal stroma (arrow).

Mucinous cystic neoplasm is histologically distinct from IPMN by a unique mesenchymal component, “ovarian type stroma,” that surrounds the cyst (Figure 11). Compared to IPMN, MCN occurs in a younger age group (mean age 55 years versus 66 for IPMN). Many cases of mucinous cystadenocarcinomas that were traditionally thought to arise from this type of tumor, likely represented invasive carcinoma arising from IPMN. Classified by the strict histologic criteria, it is extremely rare for MCN to develop invasive carcinoma (*John Hart, personal communication*). One study reported that 1 of 7 cases of MCN had an invasive carcinoma [105]. However, it is not entirely clear from this paper if this truly occurred from an MCN, as 1 of these 7 tumors exhibited communication with the main pancreatic duct and the author did not further specify. Another study examined 156 cases of MCN and found 1.3% to be invasive [106]. These probably explain the excellent overall survival associated with MCN, a 99.9% in 10 years [106]. 

## 6. Natural History of IPMN

Earlier reports had suggested a largely benign clinical course for IPMN, even in those with carcinoma *in situ* [107]. However, a definitive association between IPMN and invasive carcinoma has been established. An imaging study of patients with a PDAC and without PDAC (e.g., MRI for renal mass) showed a higher rate of IPMN in the former (7.3% versus 1.1%, with IPMN at a site distant from the PDAC) [108], suggesting a causal relationship between IPMN and some cases of PDAC. In specimens resected for IPMN, some earlier reports showed an invasive carcinoma in 53% of cases [109], a rate much higher than that in more recent series of 20–30% [110]. This is likely due to increased detection of IPMNs that resulted in more cases resected in earlier stages. Conversely, when pancreas resected for pancreatic ductal carcinomas (PDAC) are examined, 10% had been found to contain an IPMN [111]. 

Invasive carcinoma may also develop in the remnants after partial pancreatectomy for IPMN. This is likely related to the multicentricity of IPMN, leading to occult lesions left in the remnant pancreas after partial resection [112]. For main duct (MD)-IPMN with mural nodule less than 10 mm or no nodule, and with negative cytology, 10% will developed invasive carcinoma during long-term followup (a mean of 70 months) [113]. In some cases, progression to carcinoma may be extremely slow. One report described a patient who underwent resection 27 years after initial diagnosis of IPMN, without adverse event [114]. 

In general, BD-IPMNs fair better than MD- or mixed type IPMNs, particularly BD-IPMNs without symptoms [65, 110, 115, 116]. The rate of invasive carcinomas developing in patients with BD-IPMN is between 1.9 to 5.4% in long term follow up studies [116–121]. Another follow-up study of 60 patients with BD-IPMN showed a 5-year rate of 6.9% for developing PDAC [122]. Malignant progression is more closely associated with older age (70 years or older) and female gender [121]. Observed occurrence of malignancy is much faster (50% within 2 years) if pancreatitis-like symptoms are present or if the main duct is involved [123]. Others had found a lack of subsequent invasive carcinoma in large series [115, 124]. For example, in a study of 131 patients with multifocal BD-IPMN conservatively managed, no invasive carcinoma was found during a followup between 12 to 127 months [124].

 In a followup of 100 patients with BD-IPMN, mural nodule developed and reaching 1 cm in 0.62% cases per year [125]. In 5 patients with a mural nodule of 1 cm, one was found to be malignant at resection. Therefore, it is safe to follow up patient of BD-IPMN without mural nodule, or nodule less than 1 cm, without surgery [120, 125, 126].

Rare case reports had suggested a possible relationship between IPMN and pseudomyxoma peritonei (PMP) [127]. In this case, the PMP was with adenocarcinoma of omentum histologically, but without a primary carcinoma identified, except for an IPMN with mural nodule.

In summary, invasive carcinoma can be associated with IPMN in one of several ways. First, IPMN can progress from focal dysplasia to invasive disease. Second, as mentioned previously, there is always risk of subsequent malignancy if only partial pancreatectomy is performed for IPMN, owing to the multifocality of IPMN, even though additional disease foci were not evident radiologically or clinically at the time of partial pancreatectomy. Carcinoma can develop from the remnant pancreatic tissue from these unrecognized IPMN lesions. Finally, it is also possible that IPMN may be a marker for an “unstable” mucosa of the entire ductal system, which has a higher risk of malignant transformation (field effect). This scenario may account for both PDAC away from an IPMN, or PDAC develops in the remnant pancreas subsequently. Findings of multifocal discontinuous sites of dysplasia suggest the possibility of this field effect.

## 7. Clinical Outcome and Risk Factors for Invasive Carcinoma

When cases with noninvasive and invasive IPMN are assessed together, the overall 5-year survival is around 65% [128]. However, IPMN without an invasive component carry excellent prognosis [4, 7, 33, 46, 129], with 94–100% survival in some studies [5, 7, 30, 42, 66, 130, 131], and these cases have a very low recurrence rate (1.3% [129] to <8% [132]) as well, compared to those with invasive disease (50–65%) [132]. A lower 5-year survival rate of 77% for noninvasive IPMN had also been reported in individual studies [78]. In a study of 140 cases strictly limited to main duct IPMN, a 5- and 10- year survival of 100% was observed in cases without invasive carcinoma [130], compared with 60% and 50% for those with invasive carcinoma (42% of the cases). Subsequent recurrence in the remnant pancreas occurred in 8 patients, only 1 from a noninvasive case. This was found by CT scan 5 years after the initial resection. A completion pancreatectomy found a carcinoma *in situ* in the distal pancreas. 

When an invasive component is identified in IPMN, patient survival is much worse [33, 129], with reported 5-year survival rates of 24% [42], 31% [131], and 60% [130]. Many factors may account for the variation among studies, such as composition of cases at different stages of the tumor, proportion of cases with colloid carcinoma versus conventional tubular carcinoma. Case series with higher proportion of colloid carcinoma will have relatively better overall prognosis, as this type of tumor is less aggressive [79, 133]. Occasionally, a resection is performed for the preoperative diagnosis of PDAC, but in subsequent pathologic examination, a clinically inapparent BD-IPMN is identified in the area of invasive carcinoma or closely adjacent to it. Some studies may have included such cases as conventional PDACs and thus not including them as IPMN-associated carcinomas. 

Even when invasive carcinoma is present, patients with IPMN seem to have relatively better prognosis as compared to those with conventional PDAC [111, 134–136]. The 5-year survival after resection for IPMN-associated invasive adenocarcinoma is reported in another study as to be between 40% and 60% [111]. The median survival is 21 months for IPMN-associated invasive carcinoma versus 14 months for PDAC. However, the more favorable survival does not seem to stem from fundamental biological difference. IPMN-associated invasive carcinomas are usually diagnosed at an earlier stage [137]. Findings from a comprehensive single-institutional study of 1260 consecutive resections showed that IPMN-associated carcinomas are associated with lower incidence of advanced T stage, lymph node metastasis, poor tumor differentiation, or other features of aggressiveness (lymphovascular invasion, perineural invasion, positive margins), as compared to conventional PDAC [111]. If one of these adverse factors is present, the outcome is without significant difference [111]. 

In an excellent control-matched case study [79], it was found that although overall IPMN cases had a better 5-year survival, IPMN-associated tubular carcinoma cases showed no significant survival advantage as compared to conventional PDAC stage by stage. Also, when node-positive cases were compared, there is no difference in survival between IPMN-associated invasive carcinoma and conventional PDAC [136]. Others have shown the lack of difference in survival among node-positive patients between these two groups [138]. In a study with average 25.6 months followup (range 3–123 months), all cases with invasive component recurred locally or in remote sites (liver, lymph nodes, peritoneum). No patients without invasive carcinoma recurred. 

Therefore, instead of giving all patients with IPMN-associated invasive carcinoma the false hope of better prognosis, special notions should be given to the histologic tumor type. Patients with colloid carcinoma fair much better as compared to those with the tubular type carcinoma [79, 133]. 

### 7.1. Preoperative Diagnosis and Assessment for Malignancy

Preoperatively, invasion in an IPMN can be very difficult to predict [4, 9, 66, 76]. A combination of clinical features, abdominal CT, ERCP, and EUS assessment [76, 139, 140] are required in many cases, although some also advocate value of cytology evaluation [141, 142]. 

Radiologic image analyses including contrast-enhancing CT [143] and MRI are important in identifying some of the features associated with increased risk of malignancy in IPMN. A performance-comparison study of an international consensus guidelines and an 18-fluorodeoxyglucose (FDG) PET showed that while the former is more sensitive, the latter is more accurate in detecting malignant features in IPMN [139]. Current data support that FDG PET/CT may offer added value to contrast-enhancing CT for this purpose as well [143]. Some of the features that are associated malignancy and can be assessed preoperatively include main duct dilatation with a diameter of 10 mm or larger and mural nodules. The latter have a significantly higher incidence of carcinoma (86%) than those without (37%) [66, 144–146]. It must be emphasized that despite the improvement of preoperative diagnostic accuracy of imaging, a significant proportion of cases will have a change in diagnosis upon pathologic examination of resected specimen [147]. 

 Other factors believed to be important in this regard include size of pancreatic duct 6.5 mm [148] or more [144], serum carbohydrate antigen (CA) 19-9 level [148–150], and serum CEA level [145, 149, 150]. Another study did not confirm the value of CA19-9 [145]. CEA levels higher than 110 ng/mL had been shown to be highly predictive of a malignancy [145]. In addition, size of mural nodule is also related to presence of an invasive component [150]. 

A scoring system had been developed to aid in predicting malignancy preoperatively, based on analysis of 64 resected cases [148]. In this schema, size of pancreatic duct = or >6.5 mm, serum carbohydrate antigen (CA) 19-9 = or >35 U/mL scored 3 points, main duct type scored 2 points, and patulous papilla, jaundice, diabetes mellitus, and tumor size > or = 42 mm scored 1 point. Tumors with a 3 or higher point have a malignant accuracy of 90.6% [148]. Using a combination of image studies with standardized guidelines for preoperative assessment, Paye et al. found that malignant transformation can be detected with a sensitivity of 67% and specificity of 95% [76].

Based on a retrospective review of EUS-guided pancreatic fine-needle aspiration biopsy (FNAB) with histology correlation, it was noted that identification of necrosis correlated with invasive carcinoma [151]. While some studies concluded that for most cases cytologic evaluation did not offer added value on top of radiologic assessment [145], others concluded otherwise, particularly for BD-IPMN [142]. Nevertheless, there are many pitfalls in this regard and extreme caution should be exercised, as the presence of pancreatitis or nearby PanIN can lead to erroneous diagnosis of higher grade dysplasia or invasive carcinoma [152].

The International Consensus Guidelines (ICG) and several other criteria had been developed to help in clinical decision making on resection. These consensus guidelines had been shown to be sensitive in prediction for malignancy. However, the specificity is suboptimal [153, 154]. 

Overall, BD-IPMNs show less aggressive pathologic features [65]. Small BD-IPMN (less than 30 mm) without mural nodule are mostly benign [66, 77, 140, 154]. Current recommendation for resection of BD-IPMN includes cyst size ≥30 mm and mural nodules [144]. For tumor size smaller than 30 mm without symptoms or mural nodules, the patients can be safely followed [115, 155]. Some worrisome features for tumors less than 30 mm also warrant resection, including mural nodule, cyst wall thickness >2 mm, branch duct diameter >3 mm, or with main pancreatic duct involvement [146, 156]. In addition, a combination of a mural nodule >5 mm and a CEA level in the pancreatic juice >30 ng/mL is highly associated with malignancy [157]. 

### 7.2. Cyst Fluid Analysis

Chemical, immunological, or molecular analysis for cystic fluid obtained during FNA has been used by some groups in preoperative assessment, in facilitating differential diagnosis between inflammatory versus IPMN [158, 159], or benign versus malignant lesions [160]. 

Using a multiplex inflammatory mediator proteins (IMP)-targeted microarray, Lee et al. had shown that granulocyte-macrophage colony-stimulating factor (GM-CSF) and hepatocyte growth factor (HGF) detection is highly related to inflammatory cysts [159]. As discussed previously, the levels of CEA [157], CA19.9, and CA72.4 had been shown to be significantly different between benign and malignant IPMN [160]. S100A11 and S100P had been found to be increased in tissue or cyst fluid of PDAC and IPMN [93, 94].

Measurement of mRNA for SHH expression in pancreatic juice may help distinguish IPMN from chronic pancreatitis, but cannot be used to distinguish PDAC from IPMN [51]. Mesothelin mRNA level in pure pancreatic juice by RT-PCR was found in 11 (52%) of 21 pancreatic carcinomas, 5 (45%) of 11 IPMNs, and 3 (14%) of 22 with chronic pancreatitis [158]. Mesothelin expression was identified in pancreatic juice in about 45% of ductal carcinoma or IPMN, but 14% in chronic pancreatitis (by RT-PCR for mRNA) [158]. Cystic fluid prostaglandin E2 (PGE2) increases in level with grade of dysplasia, as suggested by one study [161]. 

It should be noted that currently no widely accepted modality has been established in terms of cyst fluid analysis. Many of the published assays need independent validation, and standardization of reference value needs to be developed, before they can be widely accepted as having clinical relevance. 

## 8. Treatment

Curative treatment of IPMN can be achieved by resection, although patients with small and asymptomatic IPMN without certain risk factors can be followed closely, with periodic imaging studies. Different resection modalities are performed depending on the location and size of the tumor and other clinical features. These include total pancreatectomy, pancreaticoduodenectomy, distal pancreatectomy [162, 163], central pancreatectomy, enucleation, and middle-segment-preserving pancreatectomy [164]. 

The most commonly performed procedures are pancreaticoduodenectomy (Whipple) and distal pancreatectomy with or without splenectomy. Total pancreatectomy had been performed for cases with massive involvement or disease recurrence in remnant pancreas [165]. Although this eliminates the chance of recurrent disease completely, significant complications, including infection, hypoglycemic attacks, severe diabetes, and fatty liver, often occur [165]. Medial pancreatectomy is performed for the purpose of preserving endocrine function, in which the right remnant is sutured and the left remnant anastomosed to a jejunal loop or stomach. However, the is a higher associated risk of pancreatic fistula formation (30%) [166]. Rarely, invasive carcinoma can occur from a previously unrecognized IPMN and involve the anastomosed stomach, preventing early detection (author's observation). Newer methods of function preserving, minimally invasive had been tried by some for small BD-IPMN, such as laparoscopic single-branch resection [167].

For patients who are poor operative candidates, photodynamic therapy (PDT) through ERCP had been tried for ablation of main duct IPMN [168], with good tolerance and relief of symptoms. Metastatic carcinoma occurred 2 years later in this patient [168].

By applying a guideline for resecting BD-IPMN greater than 30 mm and those less than 30 mm but with worrisome features, all the high risk lesions would have been resected and the nonresected lesions will all be of low-risk lesions (no high grade dysplasia or invasion) [169]. However, this guideline has a positive predictive value of 21.7%, leading to many low-risk cases being resected [169]. As previously mentioned, worrisome features include mural nodule, cyst wall thickness >2 mm, BD diameter >3 cm, or main pancreatic duct involvement [156]. Long-term frequent followup by ultrasound is used for lesions that show low risk features, based on size, growth rate, and most show no significant changes, with a minority of cases undergoing subsequent surgery [170].

Since many cases may have concurrent or subsequent multifocal disease, lifelong surveillance is warranted after partial pancreatectomy [64]. For IPMN without invasion, 10% may have disease recurrence after partial pancreatectomy, and no recurrence after total pancreatectomy [131]. Therefore, even for IPMN with negative resection margins, careful long-term surveillance is warranted [131].

For IPMN with invasive carcinomas, adjuvant treatment is offered to some patients, but clear benefit has yet to be established. Median survival in node-positive or node-negative cases was not improved by adjuvant treatment in one study [171]. But re-resection did show favorable outcome for recurrent carcinoma in the remnant pancreas. 

### 8.1. Intraoperative Margin Status

While intraoperative frozen section is useful in ensuring a clear margin for IPMN without an invasive component [134], recurrence is constant in cases of invasive carcinoma (10 cases) regardless of margin status [76]. The value of intraoperative frozen section for margin is thus debatable, as even a clear margin does not exclude recurrence completely. It appears that it is more critical to perform extensive sampling of the specimen in pathologic examination to exclude any focus of invasion, and margin status is only critical when only IPMN is present. Although some studies did conclude that for IPMNs with no invasion frozen section for negative margin is beneficial [134], exclusion of an invasive component can be only achieved after extensive histologic assessment in permanent sections, thus defeating the purpose. If frozen section is indeed performed, identifying a significant lesion (dysplasia in main duct) usually results in extended resection [172]. 

## 9. IPMN of Bile Duct

Tumors similar to IPMN have been seen in the bile duct as well [173], which causes abdominal pain and acute cholangitis. Current evidence seem to support the notion that papillary tumors of the biliary tree share many biological and morphologic similarities with IPMN [174], and are thus not a fundamentally different “species.” Contrary to the common assumption that these tumors arise from biliary epithelium, many cases of bile duct papillary tumors exhibit expression of MUC2, CDX2, and cytokeratin 20 [174], as they can also have pancreaticobiliary, gastric, and intestinal subtypes. Clearly, further studies of large number of cases are needed, for better understanding of these tumors.

## 10. Summary

Intraductal papillary mucinous neoplasm (IPMN) as a clinicopathologic entity encompasses a spectrum of benign cystic tumors of the pancreatic ducts. Some of these tumors progress to invasive adenocarcinomas. There are two main anatomic types, namely, main-duct IPMN and branch duct-IPMN, as determined by the region of the duct system involved. Several histomorphologic variants (or subtypes) are recognized, although the clinical significance of this “subtyping” is not well defined. Most of the asymptomatic branch-duct (BD)-IPMNs can be safely followed up, while curative resection is required for the main-duct (MD)-IPMNs. Although mucinous type carcinomas arising from IPMNs are less aggressive, carcinomas of the usual tubular type behave similarly to the conventional pancreatic duct adenocarcinomas.

## Abbreviations

CGH: Comparative genomic hybridization
DMBT1: Deleted in malignant brain tumor 1
FAL: Fractional allelic loss
FAP: Familial adenomatous polyposis
IPMN: Intraductal papillary mucinous neoplasm
IPMT: Intraductal papillary mucinous tumor
MCN: Mucinous cystic neoplasm
PanIN: Pancreatic intraepithelial neoplasia
PDAC: Pancreatic ductal adenocarcinoma
PIK3CA: Phosphoinositide-3-kinase catalytic-alpha
PJS: Peutz-Jeghers syndrome
TGM2: Tissue transglutaminase 2.
